# Cost-effectiveness analysis of alternative cooling strategies following cardiac arrest

**DOI:** 10.1186/s40064-015-1199-9

**Published:** 2015-08-19

**Authors:** Robert J Gajarski, Kurtis Smitko, Renee Despres, Jeff Meden, David W Hutton

**Affiliations:** University of Michigan Congenital Heart Center, C.S. Mott Children’s Hospital, 1540 E. Medical Center Dr. Floor 11, Rm 715Z, Ann Arbor, MI 48109 USA; Department of Health Management and Policy, School of Public Health, University of Michigan, Ann Arbor, USA

**Keywords:** Cardiac arrest, Therapeutic hypothermia, Neurologic outcome, Cost effectiveness, Decision analysis, Markov modeling

## Abstract

**Objectives:**

Using survival and neurologic outcome as endpoints , this study explored the incremental cost effectiveness of three mutually exclusive cooling strategies employed after resuscitated out-of-hospital cardiac arrests.

**Design:**

Economic analysis based on retrospective data collection and Markov modeling.

**Setting:**

Modeling based on patients housed in a tertiary ICU setting.

**Patients:**

Patients >18 years following resuscitation from out-of-hospital cardiac arrest.

**Interventions:**

Therapeutic cooling vs. conventional care.

**Measurements and main results:**

Using societal-based analytic decision modeling with a lifetime study horizon, incremental cost effectiveness ratios (ICERs) for blanket, peritoneal lavage, and V–V ECMO cooling strategies were compared with conventional care. Comprehensive cost data were obtained from available literature, national and local databases; health utility data were abstracted from previous publications and converted to quality-adjusted life years (QALYs)/person and stratified by neurologic outcome state. Future costs were discounted using a standard 3% discount rate. Cooling blankets produced better overall health outcomes at a lower cost than conventional care and V–V ECMO. Peritoneal lavage added an additional 0.67 QALYs at an ICER of $58,329/QALY. Monte-Carlo simulations incorporating uncertainty in all parameters showed that peritoneal lavage was 70% likely to be the preferred, cost-effective therapy if one were willing to pay (WTP) $100,000/QALY.

**Conclusions:**

This analysis suggests that blankets are the most cost effective cooling strategy for post-ROSC therapeutic hypothermia, with peritoneal lavage as an acceptable alternative at higher WTP thresholds. Though uncertainty about the optimal therapy could be reduced with additional research, these results can inform policy-makers and healthcare providers about cost effectiveness of alternative cooling modalities designed to improve neurologic outcome for this expanding patient population. This may be particularly relevant as societal-based cost effectiveness analyses become more widely incorporated into studies evaluating treatment for frequently encountered diseases.

## Background

Out-of-hospital cardiac arrests are relatively common with an estimated annual incidence of 50–190 events/100,000 people (Rea et al. 2004). Among these, return of spontaneous circulation varies from 10 to 40%, and in those with return of spontaneous circulation (ROSC), long-term survival is low and morbidity is high with >50% sustaining permanent neurologic injury often necessitating costly long-term care (Finn et al. 2001; Mayer 2002; McNally et al. 2011).

Therapies to restore acceptable cognitive function after anoxic brain injury associated with cardiac arrest have been disappointing, and treatment options for these patients have generally been limited to supportive care (Jastremski et al. 1989). However, several investigations using therapeutic hypothermia suggest that this therapy may provide additive neuroprotective benefits by reducing cerebral oxygen demand, edema-related intracranial pressure elevations, and the deleterious effects of free radical production associated with reperfusion following ROSC (Busto et al 1989; Morimoto et al. 1993; Bernard 1996). These lab-based findings were clinically corroborated by the Hypothermia After Cardiac Arrest (HACA) study group and a recent Cochrane analysis, both of which found that early initiation of mild-moderate hypothermia (32–34°C) was associated with improved survival and neurologic outcome (Bernard et al. 2002; Arrich et al 2012). Similarly, a single-center retrospective study of 100 non-shockable post-cardiac arrest patients found that nearly 30% of patients treated with hypothermia had a good neurologic outcome, compared with only 10% of those receiving conventional supportive care (Lundbye et al. 2012). Significantly better outcomes were noted in two recent prospective studies with findings that 90–95% of patients randomized to hypothermia vs. 70–75% of control group patients had good neurologic outcomes [Cerebral Performance Category (CPC) scores 1–2] (Safar 1981), while the remainder had poor outcomes with CPC scores 3–4 (5 = dead) (Bro-Jeppensen et al. 2009; Tiainen et al 2007; Cronberg et al. 2009). Finally, a 2013 study found that cooling, even if intended only to prevent post-arrest hyperthermia, resulted in improved neurologic outcomes (Nielsen et al. 2013).

Despite these favorable findings, cooling as a management strategy for post-resuscitation cardiac arrest has not been fully implemented in US hospitals. Lack of uptake is likely multifactorial, but cost may be a relevant contributor. Given that there are substantial costs associated with this therapy, few who ultimately survive, and even fewer who survive with intact neurological function, it could be argued that these financial resources might be better used elsewhere.

Cost-effectiveness analysis can help determine whether the costs of this therapy are justified given the outcomes associated with its use. In the only cost-effectiveness analysis thus far published on this intervention, Merchant and colleagues found that, compared with conventional care, hypothermia induced via blanket cooling improved outcomes with a cost-effectiveness ratio (~$47K/QALY) comparable to other economically acceptable US health-care interventions (Merchant et al. 2009).

In addition to blanket cooling, alternative methods to induce cooling have also been studied. These include the use of peritoneal dialysis with iced-saline dialysate and veno-venous cooling using mechanical extracorporeal support (V–V ECMO) to promote more rapid and homogenous cooling (Hoedemaekers et al. 2007; de Waard et al. 2013; Knapik et al. 2011; Testori et al 2013) Limited data on outcomes associated with these cooling modalities has been published, but the extent to which these alternative strategies may or may not be incrementally more cost effective over conventional blanket cooling has not been thoroughly explored.

While some health care providers argue that because peritoneal dialysis and V–V ECMO are more invasive strategies, they are seldom used for cooling alone. However, others advocate that in the absence of compelling contrary data (i.e. cost), all strategies should be in the treatment armamentarium—i.e. ‘supply-sensitive’ care delivery. Thus, to help guide better informed use of these modalities, the objective for this analysis was to assess the cost effectiveness of each of these strategies based on published outcomes data compared with conventional supportive care.

## Methods

### Overview

A computer-simulated, state-transition model was developed to estimate direct costs and health utilities based on probabilistic neurologic outcomes among resuscitated sudden cardiac arrest (SCA) patients treated with therapeutic hypothermia (32–34°C) vs. conventional care alone. Three independent cooling strategies were evaluated: blanket cooling, peritoneal lavage, and veno-venous extra-corporeal membrane oxygenation (V–V ECMO). Using a lifetime study horizon, base-case costs and utilities were analyzed from a societal perspective including comprehensive direct and opportunity costs for care. To make relevant comparisons between our analyses and those of Merchant, many of our model assumptions are similar to Merchant’s and are referenced as such, where appropriate, in the tables. Costs obtained from prior publications were inflated to present values based on the medical care consumer price index inflation rate, and the net present value of future costs was determined using a standard 3% discount rate. Decision-tree analysis with Markov modeling was used to derive final probability-based cost and quality-adjusted life year (QALY) data, allowing calculation of incremental cost-effectiveness ratios (ICERs). Sensitivity analysis was then performed using ranges of selected variables to explore model robustness.

### Decision model

**Fig. 1:**
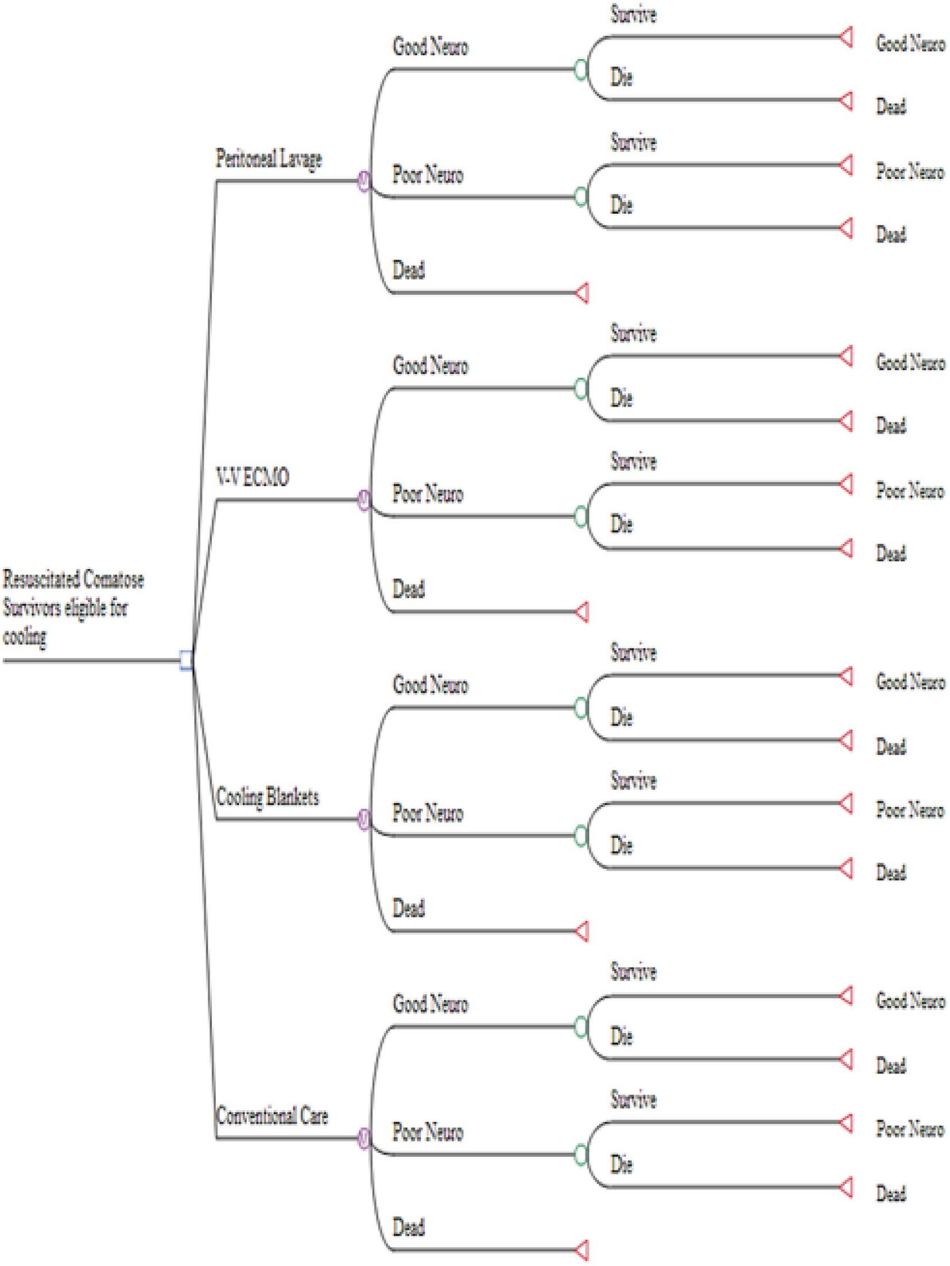
Decision tree demonstrates the potential benefit of therapeutic hypothermia given probabilities of neurologic outcome. Each enrollee is assigned to either a cooling strategy or conventional supportive care. For each decision, there is a chance probability of good, poor or dead neurologic outcome.

We structured a decision-tree analytic model to follow a hypothetical patient cohort who achieved return of spontaneous circulation (ROSC) following resuscitation from SCA (Fig. 1). We assumed that cohort enrollees, aged >18 years, met previously published inclusion criteria (Bernard et al. 2002) including short time from witnessed arrest to ROSC (<60 min). Patients were excluded if they were responding to verbal commands prior to study entry (non-comatose neurologic state does not warrant aggressive care). Other exclusion criteria were mean arterial blood pressure <60 mmHg for more than 30 min or systemic oxygen saturation <85% for more than 15 min, both of which predict poor neurologic outcome with little expected benefit from therapeutic hypothermia (Bernard et al. 2002). Probabilistic neurologic outcomes for the decision tree were determined at a 6-month interval from the SCA event and presumed to remain constant thereafter.

### Model inputs

#### Cooling approaches

Three cooling strategies were modeled: standard blanket cooling, peritoneal lavage and veno-venous extracorporeal membrane oxygenation (V–V ECMO). Blanket cooling is currently the method most frequently used in US hospitals, as it requires little training and is easily implemented. This technique requires two blankets, one placed on the anterior and one on the posterior surface of the patient, while water cooled to 32–34°C is circulated through the blanket. Blanket cooling is generally an effective method to reduce body temperature but takes several hours to accomplish. The alternative strategies are more time intensive and require more complex skill sets to implement. Peritoneal cooling is accomplished by surgically placing a dialysis catheter into the peritoneal (abdominal) cavity and instilling ice-cooled (32–34°C) saline. After 15–30 min dwell times, cooled fluid not absorbed into the vascular space through the peritoneal vessels is drained, as would be done during dialysis, and new cooled saline in infused. This process can be repeated until the desired core temperature is achieved. V–V ECMO requires surgical placement of a large-bore cannula into the central venous system. Blood is circulated through an extracorporeal circuit, which allows core temperature to be externally set. This technique requires highly skilled personnel in addition to a bedside nurse. Both of the latter approaches involve direct contact between the blood and cooling interface and, compared with blanket cooling, can induce hypothermia much more evenly and quickly. Once achieved, hypothermia is maintained for 48 h followed by passive rewarming over 8 h; peritoneal dialysis catheter and the ECMO cannula can then be removed.

#### Expected survival

Survival probabilities associated with each treatment mode (Table 1) were assigned based on previously published data (Merchant et al. 2009; de Waard et al. 2013; Testori et al. 2013). Because values for these probabilities varied due to the reference study sample size, wide ranges were documented for each variable and incorporated into the sensitivity analyses.

**Table 1:** Probabilities of Survival Based on Predicted Neurologic Outcome

	Base-Case % (range)	Reference
Probability of Death		
Conventional care	54(47–61)	[9, 13]
Hypothermia		
Blanket	50(45–54)	[9, 13, 15, 19*]
Peritoneal lavage	31(13–56)	[19*]
V-V ECMO	50(20–80)	[21]
Probability of Good Outcome Given Survival		
Conventional care	69(60–77)	[9, 13]
Hypothermia		
Blanket	86(80–90)	[9, 13, 15]
Peritoneal lavage	86(80–90)	[9, 13, 15, 19*]
V-V ECMO	75(28–97)	[21]

*V-V ECMO* veno-venous extracorporeal membrane oxygenation.

* DeWaard is the only study of peritoneal lavage, but does not report neurological outcomes in terms of fractions of patients in CPC groups. That study instead reports neurological outcomes defined in terms of average Glasgow Coma Scale of survivors. However, the average Glasgow Coma Scale results were very similar to blanket cooling, so we assume the fraction of patients in CPC groups (good outcome vs bad outcome) are similar to blanket cooling.

#### Costs

Costs for each cooling modality (Table 2) were obtained from local hospital costing databases, previously published studies, and the Healthcare Cost and Utilization Project (HCUP) database (Merchant et al 2009; National Statistics on Hospital Stays from the Healthcare Cost and Utilization Project (HCUP) 2013). Since direct HCUP costs for V–V ECMO and peritoneal lavage were not available by specific CPT code, costs were extrapolated from CCS (Clinical Classifications System) categories associated with peritoneal dialysis and extracorporeal circulatory support following cardiovascular collapse (#50 and 107, respectively). These costs were corroborated or adjusted using the University of Michigan (UM) finance department database, which provided data for each procedure. When direct costs were unavailable (detailed in Table 2), procedural charges were converted to costs using the UM critical care cost:charge ratio (0.46). We estimated length of stay based on previous publications (Merchant et al. 2009) and included in the model an average of 3 days of mechanical ventilatory ICU care (+2 days if cooled) and 18 days of floor care. Because cooling was limited to 48 h regardless of cooling strategy and catheter/cannula removal did not require additional ICU days, we felt this LOS was applicable across modalities. Daily ICU and floor care costs were obtained from the UM database. Potential complications (bleeding and infection) associated with cooling were not included in this analysis. Costs for provision of nursing care while being cooled (nursing hours and salary/nurse) were obtained from the UM financial database, and because not all centers employ therapeutic hypothermia, a cost was added to account for nursing staff training to initiate cooling. Costs for the ECMO specialists responsible for monitoring the circuit and ECMO equipment during the cooling phase were added separately. Since patients being cooled experience reflex shivering, sedation (Dexmeditomidine) and muscle relaxation (Vecuronium) is necessary; these costs were captured separately through the drug Red Book. Post-hospital costs (Table 2) including those for rehabilitation therapy, long-term care, or chronic ventilator nursing home care for those with poor neurologic outcome, were assigned based on Medical Expenditure Panel Survey (MEPS) data (2013) Duration of rehabilitation and long-term care was assigned as 30 and 365 days, respectively, based on previously published data (Merchant et al. 2009). Since all survivors with good neurologic outcome meet Class I secondary prevention indications for an implantable cardioverter-defibrillator (ICD) (Epstein et al. 2008), ICD costs for all patients in this category were included in the model. Costs for outpatient primary care follow-up including those for good and poor neurologic outcomes were captured using the MEPS database. We assumed that survivors with good neurologic outcome would need little additional caregiver care and expected that they would return to their baseline state of independence. For those with poor neurologic outcome, opportunity costs for family caregivers’ lost wages and annual median income were collected from the US Census Bureau, and patient transportation to and from follow-up appointments, were estimated using federal reimbursement/mile rates ($0.56/mile) assuming a 20 mile average round-trip travel distance.

**Table 2:** Model Input - Cost Data for Hospital and Out-Patient Care and Health Outcomes

Variable	Cost (range)	Reference
Cooling Costs ($)		
Blanket (2 blankets/person + cooling device)	6729 (4526–8951)	[17], UMHS
Peritoneal lavage	9284 (8234–9784)	[22]
V-V ECMO	39,038 (19,519–58,557)	UMHS
Hospital Care		
ICU (includes nursing costs)		
Length of stay (survivors)	5 (2–7)	[17]
Length of stay (non-survivors)	1 (0.5–3)	[17]
ICU Cost($)/day	4973 (3730–6216)	[17], UMHS
Medication (sedation) costs for cooling in ICU ($)	1197 (854–1708)	[17], UMHS, Drug Red Book
Floor (includes nursing costs)		
Length of stay	18 (10–36)	[17]
Floor cost ($)/day	2365 (1774–2956)	[17], UMHS
Post Hospital Costs ($)		
Good Neuro Outcome		
ICD (Implant and professional costs)	35,868 (26,901–44,835)	[24], UMHS
Rehabilitation therapy (Daily)	1443 (1082–1804)	[17, 23]
Rehab (days)	30 (10–90)	Assumption
Annual Ground Transportation (biannual clinic visits; reimbursement rate $0.56/mi; average 20 miles roundtrip)	22 (17–28)	GSA
Caregiver Opportunity cost (clinic visits)	175 (131–219)	US Census Bureau
Outpatient primary care (annual costs)	1305 (1180–1430)	[23]
Poor Neuro Outcome		
Outpatient primary care (annual costs)	2345 (2140–2550)	
Chronic Ventilator Care (Daily)	1582 (1187–1978)	[17, 23]
Long Term Care (Daily)	257 (193–321)	[17, 23]
Annual Ground Transportation (quarterly clinic visits;reimbursement rate $0.56/mi; 20 miles roundtrip)	44 (33–55)	GSA
Opportunity cost (lost wages-annual average)	36,410 (27,308–45,513)	US Census Bureau
Life Expectancy (years)		
Good neurologic outcome	5.5 (4–10)	[17]
Poor neurologic outcome	1.0 (0.25–3.0)	[17]
Health Utility (converted to QALY)		
Good neurologic outcome	0.76 (0.55–0.97)	[25, 26]
Poor neurologic outcome	0.35 (0.2–0.5)	[17, 26]
Discount Rate	3% (0%–5%)	Gold*

*UMHS* University of Michigan Health System, *HCUP* Healthcare Cost and Utilization Project, *MEPS* Medical Expenditure Panel Survey, *GSA* General Services Administration.

* Gold MR, Siegel JE, Russell LB, Weinstein MC, eds. Cost-Effectiveness in Health and Medicine. New York, NY: Oxford University Press; 1996.

#### Neurologic outcome

For this analysis, two mutually exclusive outcome states were used for survivors: good (CPC 1–2) and poor (CPC 3–4). Most investigators have assigned these health states based on the Cerebral Performance Categories criteria (Safar 1981). Generally, patients were assigned to an outcome state during a comprehensive neurologic assessment performed at 6 months post-SCA, (Bro-Jeppensen et al. 2009; Tiainen et al. 2007; Cronberg et al. 2009) when it was assumed that maximum improvement had occurred and a new steady state had been achieved (Table 1). Mean and ranges for each outcome state were determined based on previously published data (Lundbye et al. 2012; Bro-Jeppensen et al. 2009; Tiainen et al. 2007; Cronberg et al. 2009; Nielsen et al. 2013; Merchant et al. 2009).

### Other model assumptions

We made several additional modeling assumptions that warrant brief discussion. As mentioned above, only two neurologic outcome health states were included. We believe this is justifiable given the precedent established by previous studies, and that finer granularity of outcome state would unnecessarily increase model complexity without adding substantively to final ICER results. While other outcome criteria exist (i.e. Glasgow coma scale), we assumed, as have previous investigators, that the CPC scale is an adequately valid instrument to assess neurologic health outcomes. Additionally, we assumed that cooled patients experienced no complications. While underestimating the significance of these complications could increase cost estimates and unfavorably impact ICER estimates, because the HACA trial found no significant differences in the complication rates between those who were and were not blanket cooled, (Bernard et al. 2002) we felt this was a reasonable assumption. Finally, we included only a limited number of post-hospital discharge follow-up visits and ignored the costs of any potential new SCA-mediated morbidities requiring care or those associated with need for SCA-related hospital readmission.

### Model outputs

#### Life expectancy and QALYs

Outcome-based life expectancies were estimated using available life expectancies from previously published studies based on a patient’s probability of good or poor neurologic health state (Table 2).

Markov modeling was used to calculate total number of quality-adjusted life years (QALYs) gained. To calculate QALYs for each additional year lived, the quality of life was determined by multiplying that additional year lived by a health utility index or health-related quality of life score, thus converting that year lived into a QALY valued from 0 to 1 (how much of a year is lived in perfect health compared with an entire year in less than perfect health). Using literature-based total life expectancies for each cooling strategy or supportive care, total QALYs gained were derived for each person given their respective neurologic outcome state. Health utilities necessary to calculate QALYs were obtained from the Health-Related Quality of Life (HRQL) dataset, the Cardiac Arrest Quality of Life Database, and the Health Utilities Index for Survivors after Cardiac Arrest and based on patient CPCs (Table 2) (Nichol et al. 1999). Comparison data linking CPC scores with the Health Utilities Index for cardiac arrest survivors have been previously published (Stiell et al. 2009). Data for health utility within a given outcome state varied between cited literature, thus, values were reported with a wide range.

### Analysis

**Fig. 2:**
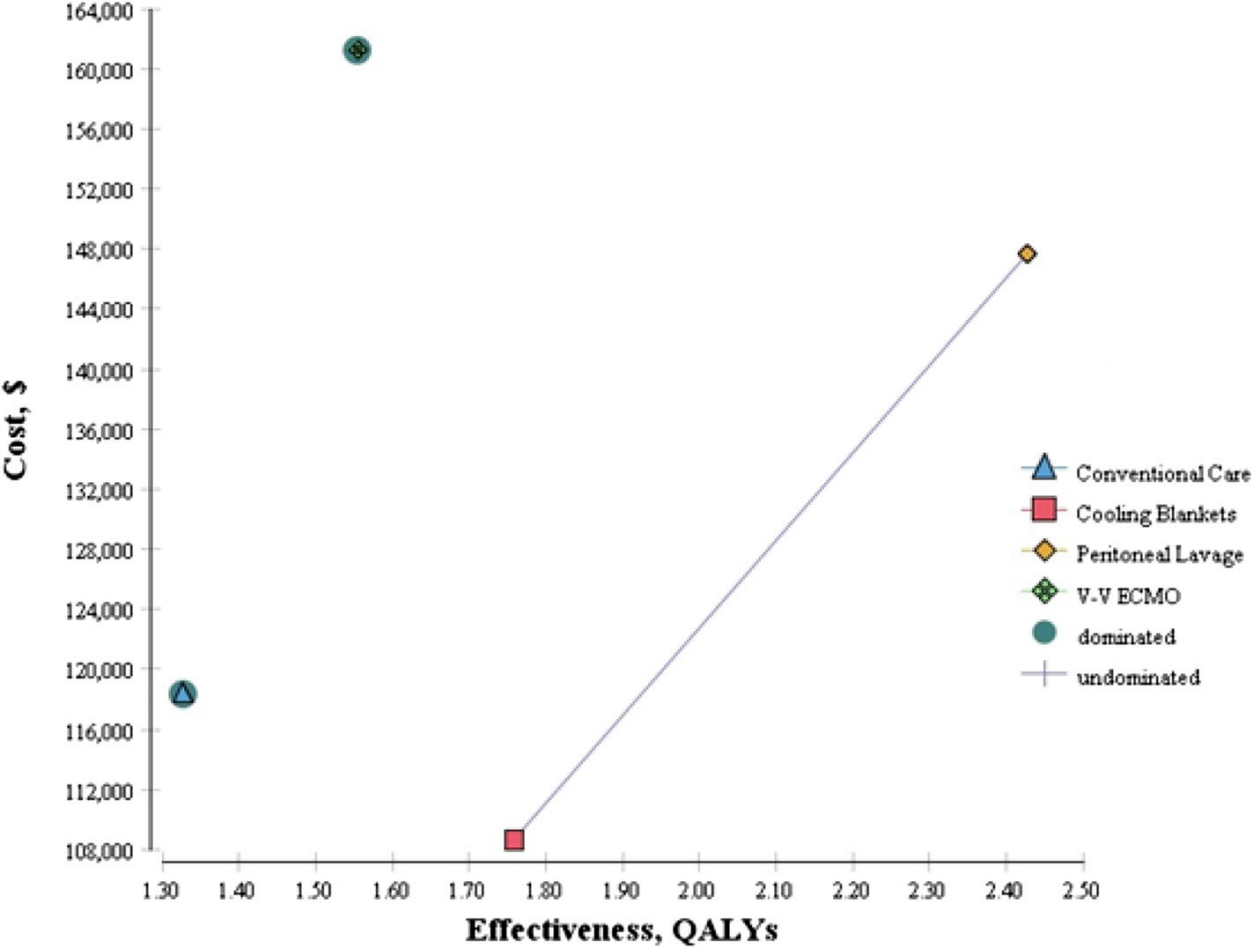
Cost effectiveness plane showing peritoneal lavage (PL) as most cost effective given WTP of ~100,000/QALY gained with cooling blanket as next best alternative. V–V ECMO is dominated by cooling blankets.

Incremental cost-effectiveness ratios (ICERs) were determined using a decision analytic modeling technique. Comparisons between each of the three cooling strategies and supportive care (shown in cost-effectiveness plane, Fig. 2) were performed as well as within cooling strategy comparisons (to test dominance). Because several of the included variables had a wide range of potential values due to differing estimates cited in previous publications, one-way, two-way, and probabilistic sensitivity analyses were performed to account for variable uncertainty and to evaluate the robustness of our model ICER estimates. The probabilistic sensitivity analysis parameterized the simultaneous uncertainty in the costs and effectiveness of each of the therapies (with uncertainty based on study sample sizes) and used Monte Carlo simulation to characterize overall uncertainty of the results. Tree Age Pro Health Care software (Tree age Software Inc^®^, Williamstown, MA, USA) was used to perform all calculations, and when needed, a willingness-to-pay (WTP) threshold of $100,000/QALY was used (Ubel et al. 2003).

## Results

### Model outputs

#### Cost outcomes

Cooling blankets had the lowest average costs at $108,640 per patient. Conventional care had the next highest costs at $118,340. Peritoneal lavage had higher costs still at $147,619. V–V ECMO costs were significantly higher at $161,226 and reflected high costs associated with a technologically advanced circulatory support system, which requires a higher skillset to initiate and maintain. For outpatient follow-up cost, ICD implantation was the major cost driver for those with good neurologic outcome as these patients met Class I ICD indications for secondary prevention. Need for long-term chronic ventilator care was the primary cost determinant for surviving patients who suffered poor neurologic outcomes.

#### Quality-adjusted life years

Using published health utility data and life expectancies stratified by neurologic outcome (Tables 1, 2), cumulative quality-adjusted life years (QALYs) were derived from the model (Table 3). Driven by the comparatively higher proportion of survivors with poor neurologic outcomes, conventional care and V–V ECMO had the lowest QALYs at 1.33 and 1.55, respectively, while blanket cooling and peritoneal lavage were associated with the highest QALYs (1.76 and 2.43). Coupled with the cost data, these point estimates of health benefit were then used to approximate base-case cost effectiveness ratios.

**Table 3:** Model Outputs—Total Costs and QALYs Gained with ICERs

Output variable	Total Costs ($) Base-Case	Total QALYs Based on Life Expectancy	Incremental Cost Effectiveness Ratio ($/QALY)
Cooling Strategy			
Cooling Blankets	$ 108,640	1.76	Reference
Conventional Care	$ 118,340	1.33	Dominated by Cooling Blankets
Peritoneal Lavage	$ 147,619	2.43	$58,329
V-V ECMO	$ 161,226	1.55	Dominated by Cooling Blankets

*QALY* Quality-Adjusted Life Year, *ICER* Incremental Cost-Effectiveness Ratio, *V-V ECMO* veno-venous extracorporeal membrane oxygenation.

#### Incremental cost effectiveness ratios

Cooling blankets were the least costly and associated with 1.76 QALYs. Conventional care and VV-ECMO were dominated by cooling blankets as they had higher costs and lower QALYs. Peritoneal lavage had an incremental cost-effectiveness ratio of $58,329 per QALY versus cooling blankets (Table 3).

#### Sensitivity analysis

**Fig. 3:**
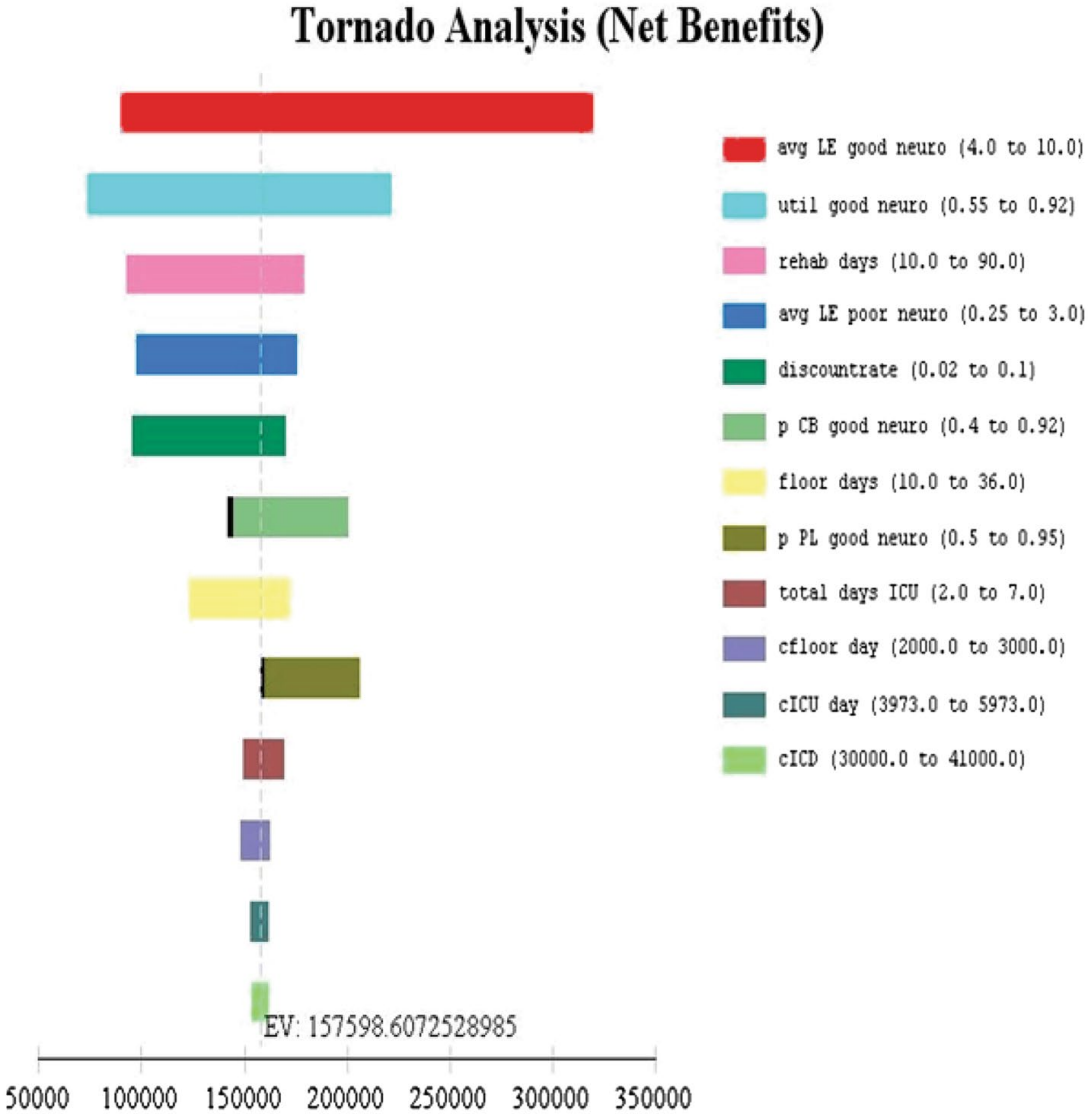
Tornado analysis demonstrating input variable impact on net benefit. Those with largest bar distribution are most influential. The *black lines* on the *bars* depicting probability of good neurologic outcome for PL and CB suggest a preference reversal for the next best alternate strategy—i.e. given a lower probability of good neurologic outcome for CB, PL would be favored and vice-a-versa based on the WTP threshold. *LE* life expectancy, *util* utility, *p* probability, *CB* cooling blanket, *PL* peritoneal lavage, *cfloor* hospital cost for 1 floor day, etc., *ICD* internal cardioverter defibrillator.

**Fig. 4:**
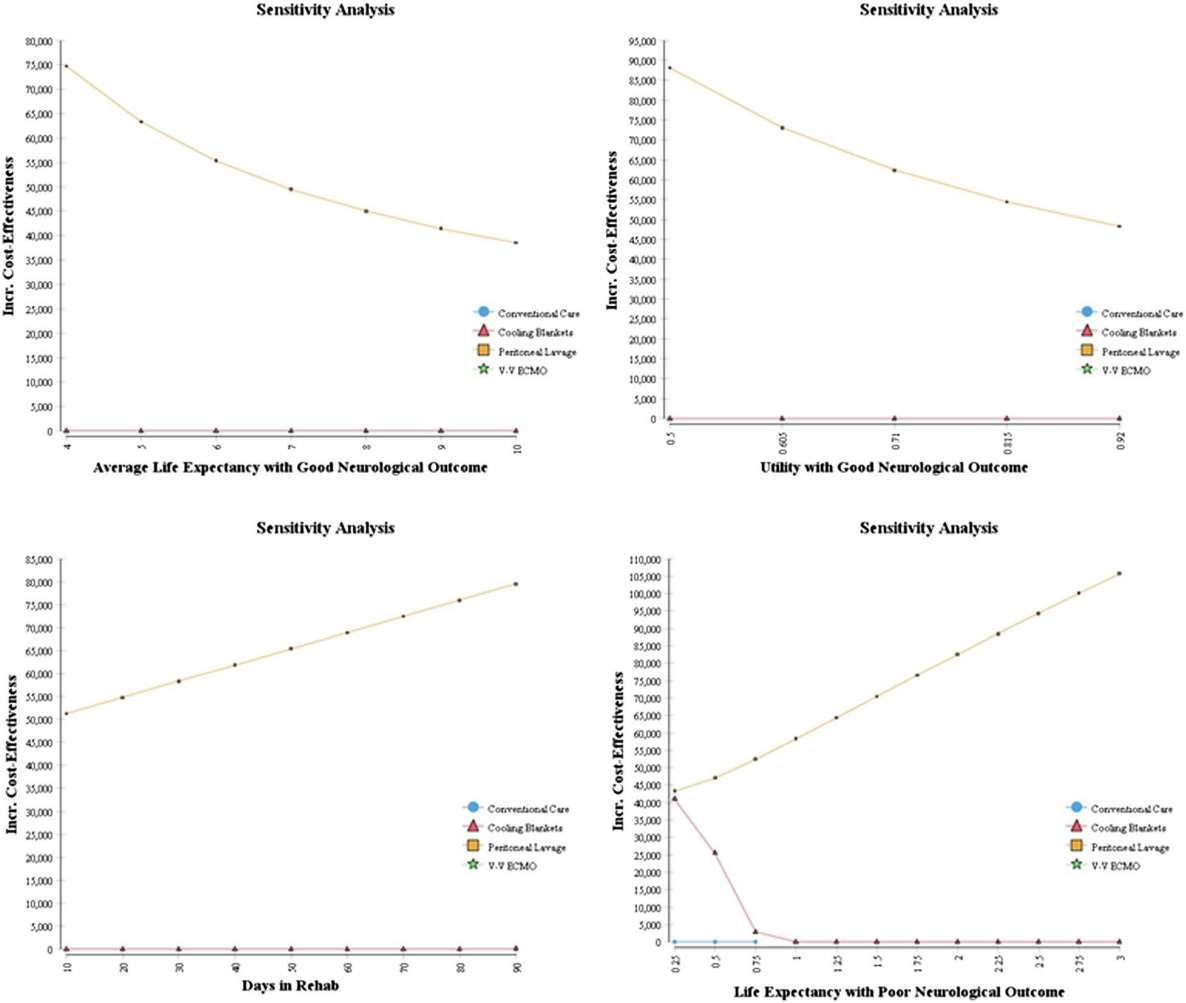
One-way sensitivity analysis of selected input variables. The Conventional Care and V–V ECMO lines are not shown as they are dominated by the other interventions. ICERs for Peritoneal Lavage are little affected and consistently remain under the WTP threshold suggesting the model analysis is robust. *le* life expectancy, *u* utility.

**Fig. 5:**
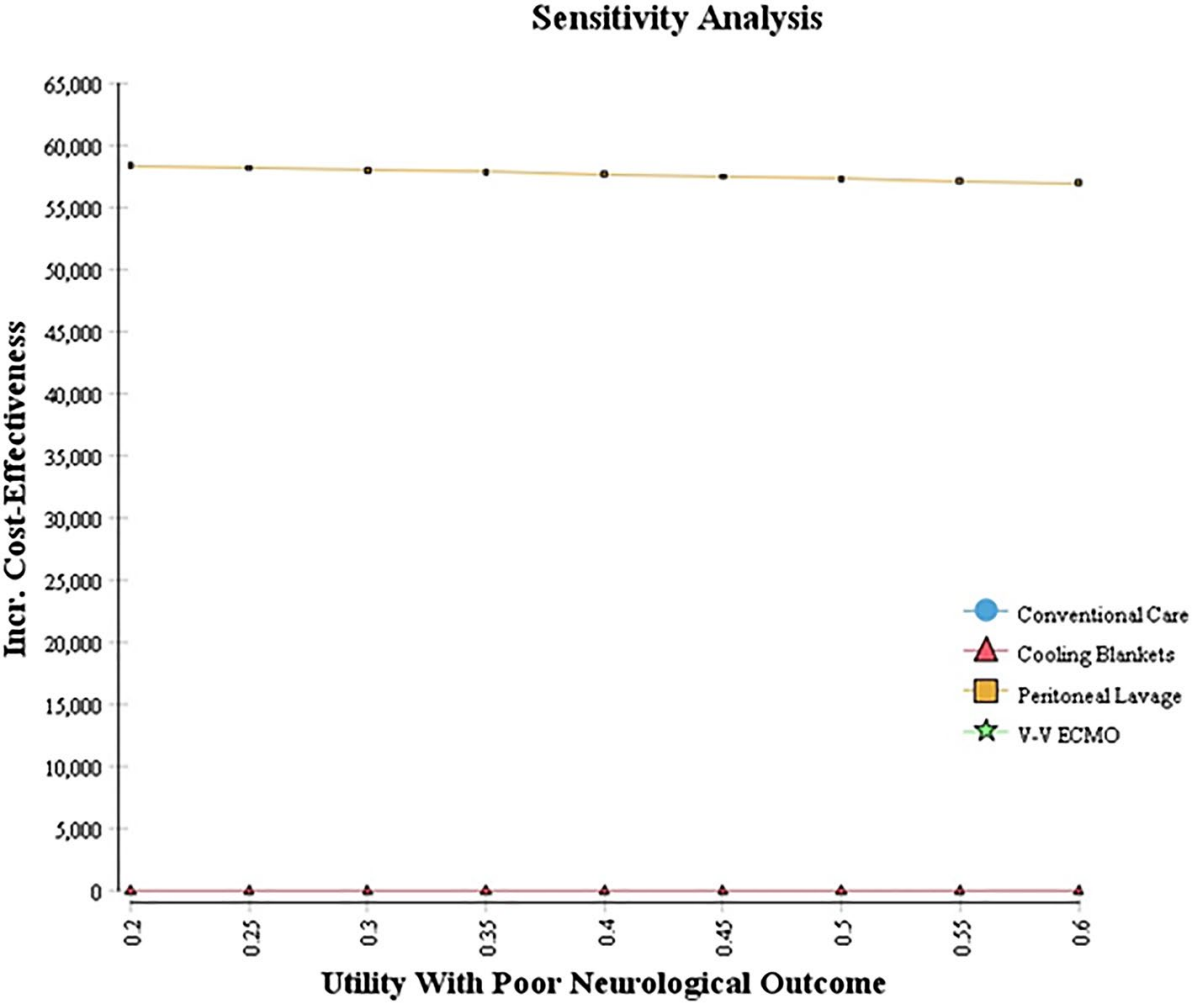
Throughout the range of utilities examined for poor neurologic outcome, the ICERs remained unchanged suggesting that our overall results are robust even if the utility among poor neurologic outcome patients were two-fold higher than our baseline estimate. V–V ECMO and Conventional Care were dominated.

**Fig. 6:**
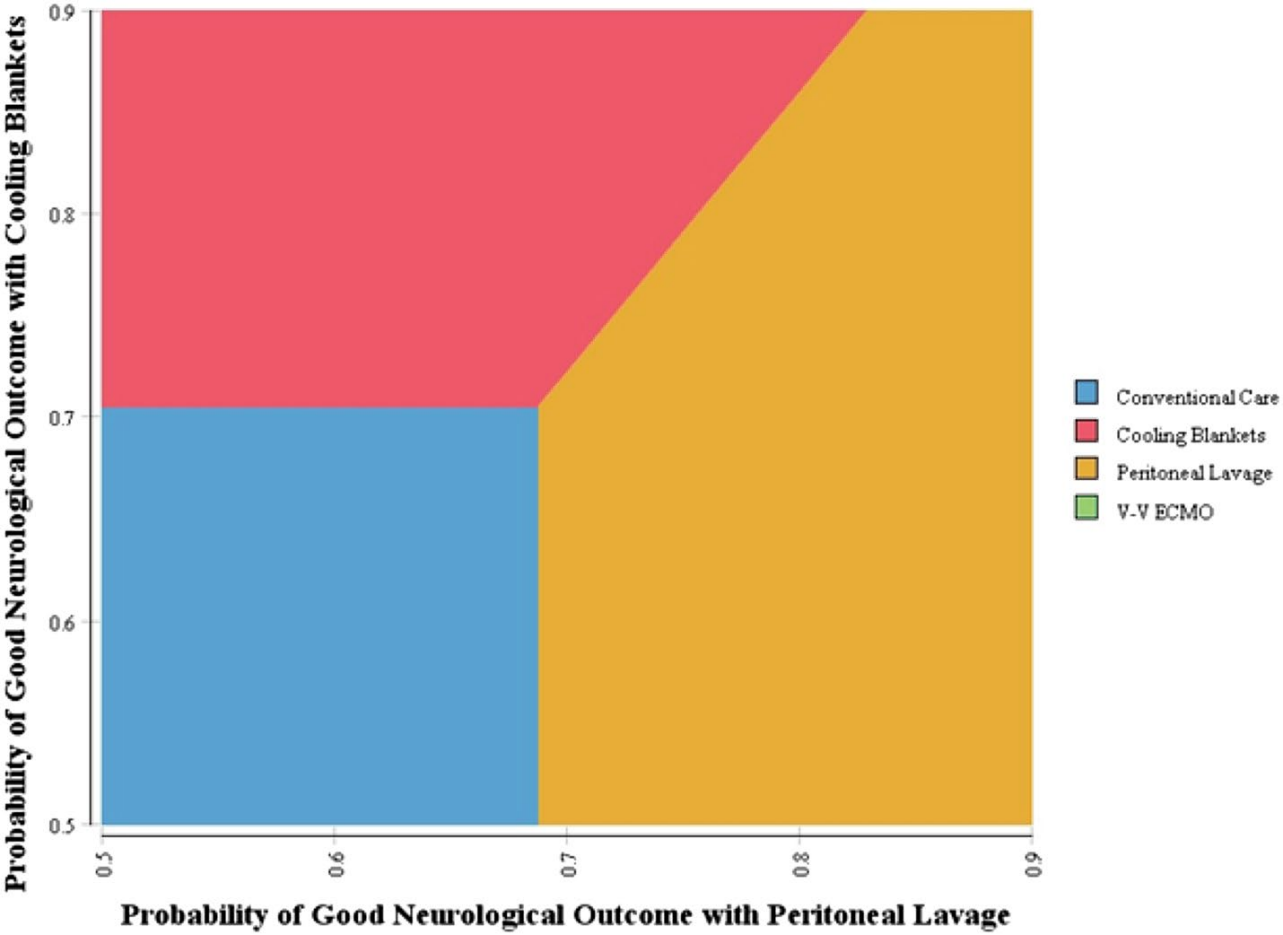
Two way sensitivity analysis showing preference for peritoneal lavage or cooling blanket based on probability of good neurologic outcome. When comparing strategies simultaneously, the one with the highest probability of good neurologic outcome will be preferred given the WTP threshold of $100,000/QALY. *p* probability, *CB* cooling blanket, *PL* peritoneal lavage, *WTP* willingness-to-pay.

As these therapies are relatively new, many parameters related to cost and efficacy are uncertain (Tables 1, 2), Tornado and one-way sensitivity analyses were performed to explore the range of net benefit for each variable (Fig. 3) and the robustness of calculated ICERs (Fig. 4). Sensitivity analyses found that the most important variable was the effectiveness of the cooling therapy at producing good neurologic outcomes (Fig. 3). Additional analyses were then performed on some of the potentially more influential variables. Since ICERs could have been affected by the assumed utility associated with poor neurologic outcome, particularly at the upper limits of our range (Table 2), a separate analysis to evaluate ICERs over a utility range varying from 0.2 to 0.6 was performed (Fig. 5). No significant change in ICERs was identified for either peritoneal lavage or cooling blankets which dominated conventional care and V–V ECMO. Furthermore, using the upper limit utility (U = 0.6) for these patients, total QALYs gained only increased 2–5% (data not shown). Finally, we assessed changes in ICER with increasing life expectancies among this group (Fig. 4, right lower panel). Given a $100K WTP threshold, peritoneal lavage and cooling blankets were acceptable cooling modalities for life expectancies under 2.75–3.0 years with cooling blankets becoming the preferred alternative beyond 3 years. Using a similar analytic strategy based on the Tornado results, we expected a reversal in preference between peritoneal lavage and cooling blankets if probability of good neurologic outcome were to change (see Fig. 3 legend). To further explore this preference reversal, a two-way sensitivity analysis was performed, which demonstrated that preference for peritoneal lavage or blanket cooling would alternate depending on the probability of good neurologic outcome for each modality (Fig. 6). Thus, between the two, the modality with the greatest probability of good neurologic outcome would be preferred based on the set WTP threshold.

#### Probabilistic sensitivity analysis

**Fig. 7:**
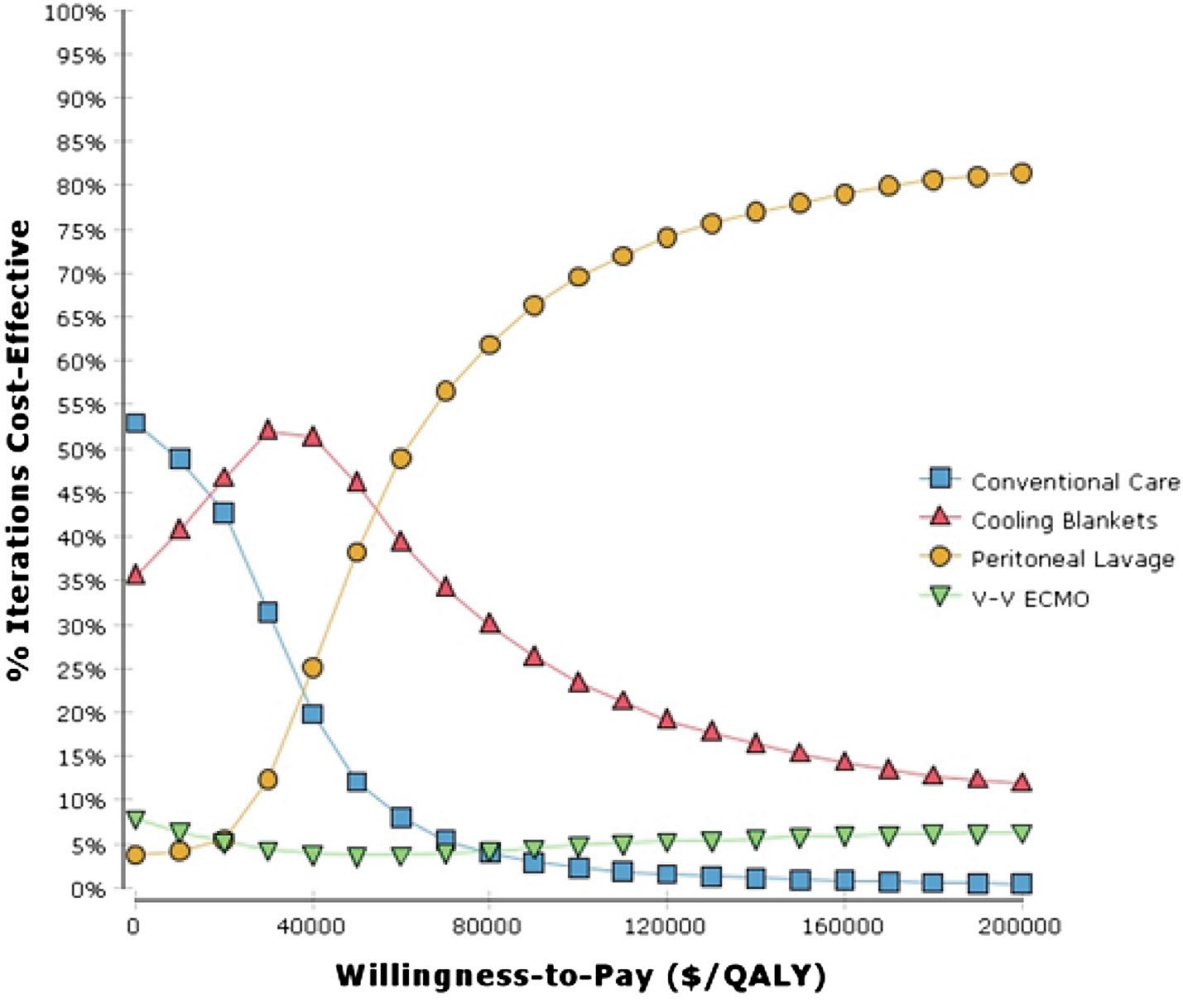
Probabilistic sensitivity analysis demonstrates the probability that a given cooling strategy will be preferred at a given willingness-to-pay level. At lower WTP thresholds, cooling blankets would be most cost effective in the majority of simulations, while at higher thresholds (>$100,000/QALY), peritoneal lavage becomes the most cost effective and preferred strategy in >70% of the simulations.

The cost-effectiveness acceptability curves (Fig. 7) show that for higher values given to a QALY, peritoneal lavage is most likely to be the cost-effective therapy of choice. At a WTP of $100,000/QALY, peritoneal lavage is 70% likely to be cost-effective. However, there is substantial uncertainty. Even at very high levels of WTP ($200,000/QALY), peritoneal lavage was preferred in only 81% of simulations. In almost 20% of simulations, another cooling modality was preferred, which reflects the substantial uncertainty regarding how effective these interventions are compared to each other.

## Discussion

Therapeutic hypothermia following resuscitated sudden cardiac arrest has been shown to improve both survival and neurological outcomes (Bernard et al. 2002; Arrich et al. 2012). The target temperature at which these benefits are best achieved is, however, unclear. A recent European study found no differences in survival or neurocognitive outcomes whether patients were cooled to 36 or 33°C. However, cooling to prevent post-arrest fevers, which increase metabolic demand and can exacerbate underlying neurologic injury, appears to be an important management strategy for these patients (Nielsen et al. 2013). The optimal modality to achieve and maintain a target temperature is difficult to determine. Given rising health-care costs and recent cost-containment efforts, particularly surrounding end-of-life resource use, cost effectiveness must be considered in selecting cooling strategies. Thus, this study used an economic decision-analytic model to examine the societal-based cost effectiveness of three alternative cooling strategies. By comparing costs and QALYs of blanket cooling, peritoneal lavage, V–V ECMO, and conventional supportive care, we found that cooling blankets and iced-saline peritoneal lavage are likely to be cost-effective strategies. We found blanket cooling to be the least expensive, but that peritoneal lavage added additional QALYs at a cost of $58,329 per QALY. The ICER of blanket cooling (vs. conventional care) was ~23K/QALY gained. These results are reasonably consistent with results cited in the only other similarly conducted cost-effectiveness analysis (Merchant et al. 2009). In that study, investigators found cooling blankets to be cost-effective versus conventional care, costing $47,168/QALY. Our finding that cooling blankets were less costly overall may be due to the inclusion of additional data on effectiveness, which lowered the likelihood of a good outcome given survival with conventional care. Our finding that PL was cost effective is due to PL having lower mortality than the other therapies and similar neurological outcomes to blanket cooling. Because of the high cost of implementing V–V ECMO and its comparative low benefit and poor survival rate, it was dominated by both blanket cooling and peritoneal lavage.

### Input variables

Multiple input variables differentially impacted model outputs. Hospital costs heavily influenced ICERs and were significantly higher for all SCA survivors. Longer stays in intensive care units (ICUs) could significantly increase costs, especially among those with poor outcome. Likewise, variations in the probability of neurologic recovery impacted cost effectiveness. In the better neurologic outcome group, for example, increased life expectancy resulted in higher total costs given the need for costly ICD implants and longer-term post-discharge clinic follow-up. However, these initial higher treatment costs were balanced by shorter hospital stays and reduced need for costly long-term institutional care which may have contributed to model favoring cooling blankets and peritoneal lavage as cost effective cooling strategies. To the contrary, survivors with poor neurologic outcome required longer hospital stays, longer rehabilitation stays, or placement in long-term care facilities. Even those who might return home would likely require chronic home care either through a home health agency with limited third party coverage or by family members who incur opportunity costs that few studies have adequately quantified. This scenario was comparatively more common among patients cooled via V–V ECMO, since the one small study of V–V ECMO used in this analysis reported a 50% mortality and poor neurologic outcome in 50% of the survivors.

### Sensitivity analysis

Although it appears likely that peritoneal lavage would be the preferred cost-effective therapy, substantial uncertainty remains about which therapy is cost-effective since the reference studies for this investigation included small sample sizes and widely variable outcomes. Specifically relevant for this analysis is the broader spectrum of potential life expectancies for those with poor neurologic outcome. Based on previous data, the present analysis assumed a 1-year total life expectancy. Since we combined CPC 3 and 4 patients into one group, longer post-arrest lifespans would have been possible for the slightly higher functioning (CPC 3) patients. To account for this, we included a broad range of life expectances for this group in our sensitivity analyses with the upper limit at 3 years, which showed no change in the model result. Since this curve was relatively flat, it is unlikely that there would have been a substantive change in data even if longer life expectancies (perhaps 4–6 years) had been included.

Our two-way sensitivity analysis showed that if cooling blankets were to have a better chance at a good neurological outcome, they would be the preferred therapy. Additional evidence for this uncertainty was shown in the probabilistic sensitivity analysis, which showed that even at high willingness-to-pay values of $100,000–$200,000/QALY (Ubel et al. 2003), there was a 20–30% chance that blankets or V–V ECMO would be the preferred therapy.

## Limitations

Our analysis has several limitations regarding the population studied and the approximation of costs and outcomes. First, this was a cohort study based on modeled data, which were not prospectively collected. The data on the effectiveness of the different cooling interventions came from different studies, and therefore may have had slightly different patient populations or levels of care. Although some cost data were captured from a local database, several costs were extrapolated from previous literature or other electronically available databases and thus subject to error. Cost errors could cause potential under- or overestimation of costs and ICERs. Secondly, in an effort to simplify the analysis, our model output may have some inherent inaccuracy related to restrictive model assumptions. We assumed only two neurologic outcome states, which did not allow for variation in long-term care costs or discrimination between CPC 3 and 4 patients, which could have impacted estimated life expectancies. Furthermore, we also assumed no complications from cooling, a fixed number of post-discharge clinic visits with no readmissions, and that co-morbidities were irrelevant to cost. Significant errors in any of these could have impacted our model results. However, given the model robustness over a wide range of variables, we have confidence in the general accuracy of our calculated ICERs. Finally, other cooling modalities such as ice packs and intravenous iced-saline fluid administration were not evaluated. In addition to the fact that cost and outcomes data were not readily available for these techniques, the consistency and efficiency with which these modalities can promote rapid cooling or maintain normothermia, which are likely critical to outcomes, cannot be guaranteed; thus assessment of these methods was not incorporated into the analysis.

## Future directions

As use of therapeutic hypothermia expands across hospital systems in the United States, additional research will be needed to monitor effectiveness and cost effectiveness of emerging cooling strategies. Additional cost-effectiveness evaluations in specific populations—for instance, pediatric patients—may also be warranted. Subgroup analyses for both pediatric and adult populations should include variables such as VF/VT vs. non-VF/VT arrest, traumatic versus medical SCA, age, ethnicity, and socioeconomic status. Likewise, additional investigations are needed to confirm the optimal timing and effect of therapeutic hypothermia on outcomes after resuscitated SCA.

If cooling does improve outcomes, health systems may consider development of EMS protocols for initiation prior to emergency department arrival. Since it is unlikely that personnel would be available to place PL catheters in the field, cooling blankets offer a feasible and acceptable alternative based on WTP criteria. If rapid deployment favorably impacts outcomes, earlier initiation of blanket cooling by trained EMS personnel could reduce total costs, increase QALYs gained, and ultimately improve cost effectiveness.

## References

[CR1] Arrich J, Holzer M, Havel C, Mullner M, Herkner H (2012) Hypothermia for neuroprotection in adults after cardiopulmonary resuscitation. Cochrane Database Syst Rev 9 (CD004128) 10.1002/14651858.CD004128.pub3 22972067

[CR2] Bernard SA (1996). Induced hypothermia in intensive care medicine. Anaesth Intensive Care.

[CR3] Bernard SA, Gray TW, Buist MD, Jones BM, Silvester W, Gutteridge G (2002). Treatment of comatose survivors of out-of-hospital cardiac arrest with induced hypothermia. N Engl J Med.

[CR4] Bro-Jeppensen J, Kjaergaard J, Horsted TI, Wanscher MC, Nielsen SL, Rasmussen LS (2009). The impact of therapeutic hypothermia on neurologic function and quality of life after cardiac arrest. Resuscitation..

[CR5] Busto R, Globus MYT, Dietrich WD (1989). Effects of mild hypothermia on ischemia-induced release of neurotransmitters and free fatty acids in rat brain. Stroke.

[CR6] Cronberg T, Lilja G, Rundgren M, Friberg H, Widner H (2009). Long-term neurological outcome after cardiac arrest and therapeutic hypothermia. Resuscitation..

[CR7] de Waard MC, Biermann H, Brinckman SL, Appelman YE, Driessen RH, Polderman KH (2013). Automated peritoneal lavage: an extremely rapid and safe way to induce hypothermia in post-resuscitation patients. Crit Care.

[CR8] Epstein AE, Di Marco JP, Ellenbogen KA, Estes M (2008). AHA guidelines for device-based therapy of cardiac rhythm abnormalities. Circulation.

[CR9] Finn JC, Jacobs IG, Holman CD, Oxer HF (2001). Outcomes of out-of-hospital cardiac arrest patients in Perth, Western Australia, 1996–1999. Resuscitation..

[CR10] Hoedemaekers CW, Ezzahti M, Gerritsen A, van der Hoeven JG (2007). Comparison of cooling methods to induce and maintain normo- and hypothermia in intensive care unit patients: a prospective intervention study. Crit Care.

[CR11] Jastremski M, Sutton-Tyrrell K, Vaagenes P (1989). Glucocorticoid treatment does not improve neurologic recovery following cardiac arrest. Brain Resuscitation Clinical Trial I Study Group. JAMA.

[CR12] Knapik P, Rychlik W, Siedy J, Nadziakiewicz P, Cieśla D (2011). Comparison of intravascular and conventional hypothermia after cardiac arrest. Kardiol Pol..

[CR13] Lundbye JB, Ramu B, Hosseini-Khalili A, Li D, Slim HB, Bhavnani SP (2012). Therapeutic hypothermia is associated with improved neurologic outcome and survival in cardiac arrest survivors of non-shockable rhythms. Resuscitation..

[CR14] Mayer SA (2002). Hypothermia for neuroprotection after cardiac arrest. Curr Neurol Neurosci Rep.

[CR15] McNally B, Robb R, Mehta M, Vellano K, Valderrama AL, Yoon PW (2011). Centers for Disease Control and Prevention. Out-of-hospital cardiac arrest surveillance—Cardiac arrest registry to enhance survival (CARES), United States, Oct 1, 2005–Dec 31, 2010. MMWR Surveill Summ.

[CR16] Medical Expenditures Panel Survey (MEPS) from the Agency for Healthcare Research and Quality. http://www.meps.ahrq.gov/mepsweb/data_stats/tables-compendia. Accessed 10 Jan 2013

[CR17] Merchant RM, Becker LB, Abella BS, Asch DA, Groeneveld PW (2009). Cost-effectiveness of therapeutic hypothermia after cardiac arrest. Circ Cardiovasc Qual Outcomes..

[CR18] Morimoto Y, Kemmotsu O, Kitami K (1993). Acute brain swelling after out-of-hospital cardiac arrest: pathogenesis and outcome. Crit Care Med.

[CR19] National Statistics on Hospital Stays from the Healthcare Cost and Utilization Project (HCUP) and Agency for Healthcare Research and Quality. http://www.hcupnet.ahrq.gov. Accessed 10 Jan 2013

[CR20] Nichol G, Stiell IG, Hebert P, Wells GA, Vandemheen K, Laupacis A (1999). What is the quality of life for survivors of cardiac arrest? A prospective study. Acad Emerg Med..

[CR21] Nielsen N, Wetterslev J, Cronberg T, Erlinge D, Gasche Y, Hassager C (2013). Targeted temperature management at 33°C versus 36°C after cardiac arrest. N Engl J Med.

[CR22] Rea TD, Pearce RM, Raghunathan TE, Lemaitre RN, Sotoodehnia N, Jouven X, Siscovick DS (2004). Incidence of out-of-hospital cardiac arrest. Am J Card..

[CR23] Safar P, Grenik A, Safar P (1981). Resuscitation after brain ischemia. Brain failure and resuscitation.

[CR24] Stiell IG, Nesbitt LP, Nichol G, Maloney J, Dreyer J, Beaudoin T (2009). Comparison of the cerebral performance category score and the health utilities index for survivors of cardiac arrest. Ann Emerg Med.

[CR25] Testori C, Holzer M, Sterz F, Stratil P, Hartner Z, Moscato F (2013). Rapid induction of mild therapeutic hypothermia by extracorporeal veno-venous blood cooling in humans. Resuscitation..

[CR26] Tiainen M, Poutiainen E, Kovala T, Takkunen O, Happola O, Rione RO (2007). Cognitive and neurological outcome of cardiac arrest survivors treated with therapeutic hypothermia. Stroke.

[CR27] Ubel PA, Hirth RA, Chernew ME, Fendrick AM (2003). What is the price of life and why doesn’t it increase at the rate of inflation*?*. Arch Intern Med.

